# Effect of Whey Protein Supplementation in Postmenopausal Women: A Systematic Review and Meta-Analysis

**DOI:** 10.3390/nu14194210

**Published:** 2022-10-10

**Authors:** Yao-Yi Kuo, Hao-Yun Chang, Yu-Chen Huang, Che-Wei Liu

**Affiliations:** 1 School of Medicine, College of Medicine, Taipei Medical University, Taipei 110, Taiwan; 2 Division of General Medicine, Department of Medical Education, Shuang Ho Hospital, Taipei Medical University, New Taipei City 235, Taiwan; 3 Division of General Medicine, Department of Medical Education, Far Eastern Memorial Hospital, New Taipei City 220, Taiwan; 4 Research Center of Big Data and Meta-Analysis, Wan Fang Hospital, Taipei Medical University, Taipei 116, Taiwan; 5 Department of Dermatology, Wan Fang Hospital, Taipei Medical University, Taipei 116, Taiwan; 6 Department of Dermatology, School of Medicine, College of Medicine, Taipei Medical University, Taipei 110, Taiwan; 7 Department of Orthopedics, Cathay General Hospital, Taipei 106, Taiwan; 8 School of Medicine, College of Medicine, Fu Jen Catholic University, New Taipei City 242, Taiwan; 9 School of Medicine, National Tsing Hua University, Hsinchu 300, Taiwan

**Keywords:** postmenopausal, resistance training, sarcopenia, whey protein, women

## Abstract

(1) Background: Whey protein (WP) in combination with resistance training (RT) is beneficial in improving sarcopenic obesity and its damaging effects in older adults, while the difference between men and women should be considered while interpreting results. This review aims to investigate WP’s efficacy on postmenopausal women with or without RT; (2) Material and Methods: We searched electronic databases including PubMed, EMBASE, and the Cochrane Library from inception to August 2021 for randomized controlled trials that included comparison groups to evaluate WP’s efficacy in women aged 55 years and above. The outcomes included body composition, muscular strength, functional capacity, and dietary intake. Standardized mean differences (SMDs) with 95% confidence intervals (CIs) were used to estimate the effect of WP. We also performed subgroup analysis with or without RT; (3) Results: We included 14 studies in the systematic review and 10 studies in the meta-analysis. Subgroup analyses showed RT was a major confounder for muscle strength, lean mass, and dietary protein intake (PI). In the RT subgroup, WP supplementation had a significant positive effect on biceps curl strength (BC) (SMD: 0.6805, 95% CI: 0.176, 1.185, *I*^2^: 0%), and lower limb lean-mass (LLLM) (SMD: 1.103, 95% CI: 0.632, 1.574, *I*^2^: 14%). In the subgroup without RT, a significant negative effect on PI (SMD: −0.4225, 95% CI: −0.774, −0.071, *I*^2^: 47%) was observed, while no significant effect on muscle strength or lean mass was revealed. WP supplementation did not show a significantly different effect on fat mass or body weight loss in both the subgroups; (4) Conclusions: In postmenopausal women, WP supplementation only in combination with RT enhances BC and LLLM compared to placebo controls. Without RT, WP has no significant benefit on muscle strength or lean mass.

## 1. Introduction

Aging is an irreversible process which predisposes individuals to many biological alterations, often associated with a progressively decreased muscle mass and strength (sarcopenia), and an increased fat mass (obesity) [1]. This combination, termed sarcopenic obesity, can cause chronic inflammation thus explaining the increased risk of developing metabolic syndrome and comorbidities, such as type-2 diabetes mellitus, dyslipidemia, and cardiovascular diseases [2,3]. Moreover, the reduction in muscular function capacity enhances the risk of falls and fractures. This reduces physical performance and causes higher mortality in older individuals [4,5]. Thus, measures to attenuate the age-related health declines and to improve the quality of life of the elderly have become a global effort.

Older women generally possess lower levels of muscle mass and muscular strength, and consume a lower baseline protein intake (PI) as compared to men [6,7]. The decrease in physical function may also contribute to a higher risk of falls. Moreover, older postmenopausal women have increased total cholesterol and LDL-c, decreased HDL-c, elevated blood pressure, and insulin resistance due to a lack of estrogen protection [8]. Hence, older women with these risks should be considered susceptible to the damaging effects of sarcopenia.

Whey protein (WP) is commonly used as protein supplementation compared to other sources of protein as it is easily digested and leads to rapid amino acid delivery to skeletal muscles, making it easier to meet the body’s protein requirements. It is rich in leucine and is therefore, considered more effective in stimulating muscle protein synthesis [9,10].

A previous prospective cohort study indicated that higher dairy PI has lesser association with low muscle mass and abdominal obesity [11]. Previous studies comparing WP and an ordinary protein-rich diet via dietary counseling demonstrated that if the amount of protein reaches a threshold (1.2–1.5 g/kg BM^−1^ day^−1^), muscle mass can be increased in sarcopenic elders. Additionally, under achieving adequate protein intake (1.2–1.5 g/kg BM^−1^ day^−1^), WP can further improve gait speed compared to the ordinary protein-rich diet group in the sarcopenic elderly [12].

The combination of resistance training (RT) and protein supplementation are the most common non-pharmacological strategies for older adults to attenuate age-related changes in metabolic and body composition [13]. RT provides mechanical stimuli that are considered effective for increasing muscle mass and strength, reducing fat mass and inflammatory biomarkers, and improving metabolic syndrome profile [14]. Moreover, functional training which uses movements similar to performing activities of daily living also improves neuromuscular qualities, e.g., balance, flexibility, and endurance, resulting in a better quality of life for the elderly [15].

The effects of protein with or without RT may differ according to the population characteristics, such as the age group, gender, and the protein chosen. The results may be confounded by the proportion of both genders included, as the differences in metabolism between men and women should be considered [16,17]. Previous studies have assessed the additive effect of higher protein consumption on RT, which showed an increase in muscle mass, strength, and improved performance in the elderly compared to control group [18,19,20,21].

WP has already demonstrated its potential role in RT within the general population. However, there is currently no consensus on the effects of protein supplementation on muscular adaptions and nutritional intake in older women. This systematic review and meta-analysis with randomized controlled trials (RCTs) was conducted to investigate the efficacy of WP and evaluate the differences between elderly women with or without RT.

## 2. Materials and Methods

This study was conducted in accordance with the 2020 Preferred Reporting Items for Systematic Reviews and Meta-Analyses guidelines (Table S1 (Supplementary Materials)), and was registered on PROSPERO (CRD42022330726).

### 2.1. Literature Search Strategy

Relevant studies were identified from the following electronic databases: PubMed, EMBASE, and the Cochrane Library from inception to August 2021 without any language restriction. The primary search terms in the title and abstract were used as follows: (whey protein) AND (menopause women) OR (older women)) for selecting eligible studies. The detailed search strings are shown in Method S1. To broaden the search scope, additional studies were obtained by screening the citations of relevant studies from “similar articles” listed in PubMed.

### 2.2. Eligibility Criteria and Study Selection

The eligible studies should meet the following inclusion criteria: (1) A randomized controlled trial that included comparison groups to evaluate the efficacy of diet supplemented with WP, either with or without RT; (2) Studies that used validated tools to evaluate body composition, lean mass (depicted as lean mass, muscle mass, or lean soft tissue mass), muscular strength, functional capacity, and dietary intake outcomes; and (3) Study participants that included women above 55 years of age. We excluded articles that met at least one of the following criteria: (1) Study types other than RCTs; (2) Non-human research; and (3) Insufficient outcome data. Titles and abstracts of potentially eligible studies were screened, their full texts were retrieved, and reviewed based on the eligibility criteria. Two independent authors (H. Y. Chang and Y. Y. Kuo) were involved in the retrieval, screening and selection process.

### 2.3. Data Extraction and Quality Assessment

Two independent authors (H. Y. Chang and Y. Y. Kuo) extracted the data from the included studies. The extracted data included the author’s surname, year of publication, country, ethnicity, sample size, participant characteristics, exercise interventions, changes between baseline and endpoint on study outcomes, study duration, and time points of data collection. The methodological quality of each included study was assessed by using the modified Cochrane Risk of Bias tool Version 2.0, which included the following five domains: allocation bias, performance bias, attrition bias, detection bias, and reporting bias. The overall bias of each article was determined to be low, unclear, or high risk. A third reviewer (Y.C. Huang) was consulted for any disagreement to reach a consensus.

### 2.4. Data Synthesis and Statistical Analysis

The primary outcomes were changes in muscular strength and functional capacity, and gain of lean mass; the secondary outcomes were changes in fat mass, loss of body weight, and dietary intake from baseline study outcome to the treatment endpoint. Meta-analyses were performed when at least two studies compared the same outcome in participants receiving or not receiving WP supplementation. A subgroup analysis for participants with or without RT was also performed. Continuous variables were expressed by standardized mean differences (SMDs) with 95% confidence intervals (CIs). A random-effects model was adopted due to possible heterogeneity. Data heterogeneity was assessed using the Cochran Q-test and *I*^2^ test. Both funnel plot and Egger’s test were used to assess publication bias if more than 10 studies were available [22]. All of the analyses were performed using Comprehensive Meta-Analysis Version 3 software (Biostat, Inc., Englewood, NJ, USA).

### 2.5. Quality of Evidence

The Grading of Recommendations, Assessment, Development, and Evaluations (GRADE) approach including risk of bias, inconsistency, indirectness, imprecision, and publication bias was used to evaluate the quality of evidence of each outcome in this systematic review and meta-analysis.

## 3. Results

### 3.1. Search Results and Trial Characteristics

**Figure 1:**
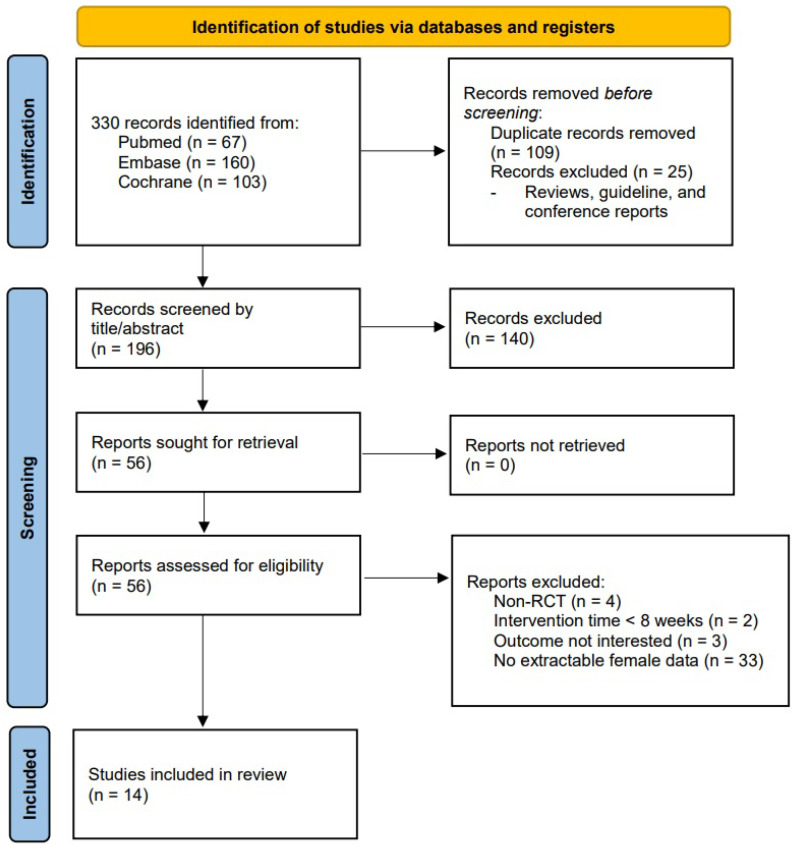
Flow diagram. Flow diagram of the literature search and selection process.

The primary search terms initially identified 330 records potentially eligible for inclusion. After excluding 109 duplicated records along with 25 records that were reviewed, guidelines, or conference reports, a total of 196 records entered the screening stage. After assessing the title or abstract, 140 records were excluded as they were irrelevant to our research, resulting in a total of 56 reports that were sought for retrieval and assessed for eligibility. Forty-two reports were excluded as they met the exclusion criteria or did not meeting the inclusion criteria. Fourteen reports were included in the review (Figure 1).

All 14 included studies were RCT with a control group. Among these, 2 were single blinded [23,24], and 12 were double blinded [25,26,27,28,29,30,31,32,33,34,35,36]. These studies were implemented in six different countries with the majority having an intervention duration of 12 weeks (ranged from 10 weeks to 72 weeks). Detailed study characteristics are shown in Table 1 and Table 2. Among these 14 studies, 11 studies included the data of WP with RT [23,24,25,26,27,28,29,30,31,32,35], and 4 studies included the data of WP without RT [23,33,34,36]. Furthermore, seven studies were conducted by the same team and the participants were from the same group [26,27,28,29,30,31,32]. To address the concern of bias due to overlapped participants, we only selected one study with the largest number of participants among these seven studies for each outcome. In addition, another two studies were conducted by the same team with the same participants [34,36]. However, they did not present the same outcomes and we included both of them in our study. We reported each study rather than collapsing to one to clarify the difference between these studies. Finally, four studies were excluded in the meta-analysis for no outcome selected [26,28,29,32], while 10 studies were included in the meta-analysis for quantitative analysis [23,24,25,27,30,31,33,34,35,36].

### 3.2. Risk of Bias Assessment

**Figure 2:**
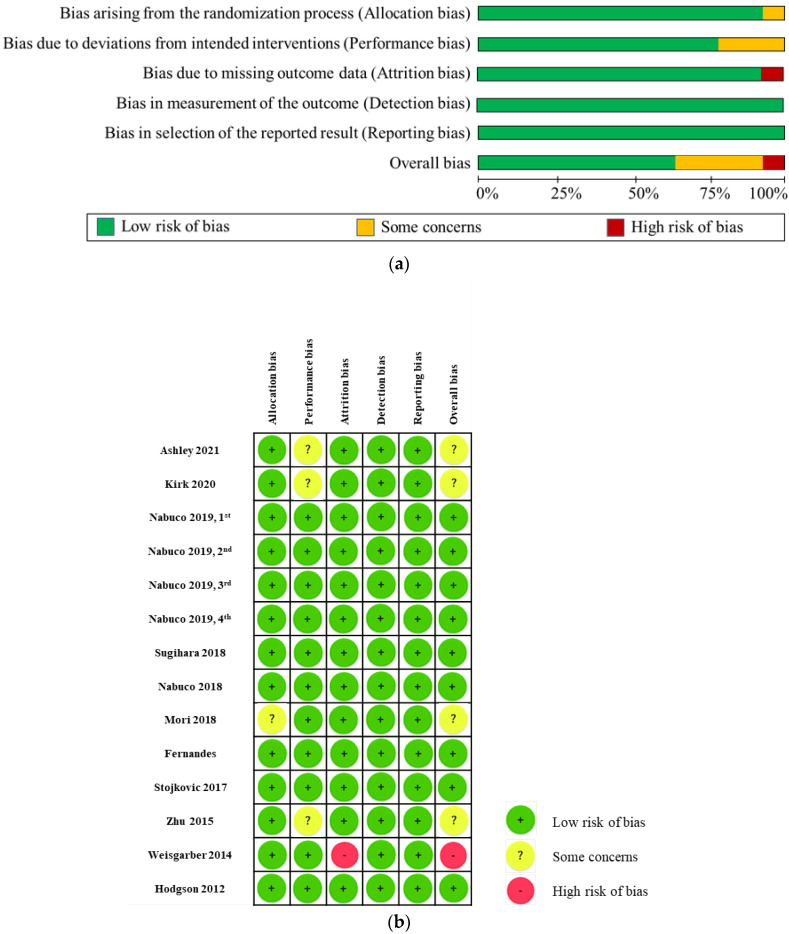
(**a**). Risk of bias assessment. Presented as a percentage across all included studies; (**b**). Risk of bias assessment. For all included studies [24,25,26,27,28,29,30,31,32,33,34,35,36].

The risk of bias assessment of the studies included in this systematic review is presented in Figure 2a,b. High risk of bias was only seen in the domain of attrition bias in one study for incomplete outcome data [35]. In that study, 12 participants completed the study while the outcome data of biceps curl strength (BC), knee extension strength, and knee flexion strength were available from only 10 of them. There was no evidence that the result was unbiased, and the missing data likely depended on true value. Therefore, attrition bias in this study was rated as high.

### 3.3. Participants’ Characteristics

The total participants across the included studies were 776, with average age ranging from 57 to 74 years and average BMI ranging from 22 to 28 kg/m^2^. Participants were declared to be “healthy” in four studies [23,24,25,35], and to be “healthy or sarcopenic” in three studies [27,30,31]. Participants were classified to be PI deficient in two studies, reporting a PI at baseline lower than the current adult recommended dietary-allowances [30,32].

### 3.4. RT Characteristics

Among the 10 included studies, 7 studies included the data of WP with RT. The RT interventions duration ranged from 10 to 24 weeks undergoing RT for 2 to 3 days per week, with 4–8 exercises per session, with 0–6 sets per exercise, and with 8–12 repetitions per set (or to fatigue). All the studies involved upper and lower body RT. Each outcome of muscle strength was trained by specific RT programs in all the studies.

### 3.5. WP Characteristics

Among the 10 included studies, 9 studies orally gave a range of 20 to 40 g of daily protein supplement [24,25,27,30,31,33,34,35,36]. Eight studies used WP [23,24,25,27,30,31,33,35], and two studies used skimmed milk plus WP isolate [34,36]. Furthermore, six studies provided protein supplements after RT in addition to regular meals on no RT days [24,25,27,30,31,35]. Four studies provided protein supplements daily in addition to regular meals [23,33,34,36]. All control groups did not receive protein supplementation. Eight studies used placebo supplements mainly consisting of carbohydrates with similar flavor [25,27,30,31,33,34,35,36]. Among them, seven studies used placebo supplements with similar calories content [27,30,31,33,34,35,36]. Two studies did not use placebo supplements [23,24].

### 3.6. Results of Meta-Analysis

The detailed data of each included study are shown in Tables S2–S5. The results of the meta-analysis are shown in Table 3.

### 3.7. Effect of WP Supplementation on Muscle Strength and Functional Capacity

We included knee flexion strength (KF), grip strength (GS), knee extension strength (KE), chest press (CP), gait speed test (GST), biceps curl strength (BC), and rising from sitting position (RFSP) in the muscle strength and functional capacity assessment. The participants with WP supplementation showed no significant difference in the change of KF (SMD: 0.044, 95% CI: −0.214, 0.302,), GS (SMD: 0.101, 95% CI: −0.107, 0.308,), KE (SMD: 0.027, 95% CI: −0.194, 0.248), and RFSP (SMD: −0.036, 95% CI: −0.291, 0.219), compared to those without WP supplementation. (Figures S1–S7)

In the RT subgroup, the pooled estimate revealed, the participants receiving WP had a significant increase in BC (SMD: 0.6805, 95% CI: 0.176, 1.185) than those without WP. There was no difference in the change of KF (SMD: 0.2905, 95% CI: −0.756, 1.337), GS (SMD: 0.1765, 95% CI: −0.156, 0.509), KE (SMD: 0.364, 95% CI: −0.031, 0.759), CP (SMD: 0.108, 95% CI: −0.268, 0.484), and GST (SMD: 0.1625, 95% CI: −0.261, 0.586).

Meta-analyses of CP, BC, and GST were not performed in the WP group without RT due to lack of data availability. The results of WP group without RT revealed that participants receiving WP showed no significant change of KF (SMD: 0.0285, 95% CI: −0.238, 0.295), GS (SMD: 0.0515, 95% CI: −0.215, 0.318,), and KE (SMD: −0.1255, 95% CI: −0.392, 0.141).

### 3.8. Effect of WP Supplementation on Muscle Mass Gains

We included upper-limb lean mass (ULLM), lower-limb lean mass (LLLM), and skeletal muscle mass (SMM) in the muscle mass gain-assessment. The results showed that WP supplementation may not enhance the outcomes related to muscle mass. A significant negative effect was demonstrated in ULLM (SMD: 0.2415, 95% CI: −0.473, −0.01), while that of LLLM (SMD: 0.028, 95% CI: −0.214, 0.27) and SMM (SMD: 0.080, 95% CI: −0.533, 0.693) did not show a significant difference (Figures S8–S10).

In the RT subgroup, WP supplementation may enhance all of the outcomes related to muscle mass except for ULLM, which showed no effect. Moreover, a significant effect was demonstrated only in LLLM (SMD: 1.103, 95% CI: 0.632, 1.574), while no significant effect was shown in ULLM (SMD: 0, 95% CI: −0.405, 0.405,), and SMM (SMD: 0.4775, 95% CI: −0.473, 1.428).

Meta-analyses of ULLM, LLLM and SMM were not performed in the WP group without RT due to the lack of data availability.

### 3.9. Effect of WP Supplementation on Fat Mass (FM) and Body Weight Loss (BW)

The participants with WP supplementation showed an increase in FM and BW loss relative to the PLA groups, while no significant effect was demonstrated, including FM (SMD: −0.107,95% CI: −0.443, 0.229) and BW (SMD: −0.115, 95% CI: −0.291, 0.061). (Figures S11 and S12)

In the RT subgroup, the pooled estimate revealed that participants receiving WP showed an increase in FM loss (SMD: −0.0885, 95% CI: −0.427, 0.25), and BW loss (SMD: −0.0205, 95% CI: −0.386, 0.345) relative to the PLA groups without significant difference.

In the subgroup of participants without RT, the results revealed that WP supplementation showed an increase in FM loss (SMD: −1.2825, 95% CI: −3.975, 1.410), and BW loss (SMD: −0.1435, 95% CI: −0.344, 0.057) relative to the PLA groups without significant difference.

### 3.10. Effect of WP Supplementation on Daily Dietary Nutrients Intake

We regarded daily dietary intake as an outcome in our study to investigate how the introduction of a daily supplement impacted on daily macronutrient and energy intakes. We included total energy intake (TEI), carbohydrate intake (CHI), protein intake (PI), and fat intake (FI) in daily dietary nutrient intake assessment. We used the dietary nutrient intake with deduction of the WP and placebo supplementation to render the results more objective. Two studies presented total nutrient intake including WP and placebo supplementation [30,36], while they both provided the nutrient composition of the supplements. Therefore, the dietary nutrient intake with WP and the placebo supplementation deduction were calculated according to the supplementation protocol.

The participants with WP supplementation showed that WP supplementation may reduce PI (SMD: −0.0685, 95% CI: −0.396, 0.259), TEI (SMD: −0.067, 95% CI: −0.293, 0.159), CHI (SMD: −0.0205, 95% CI: −0.365, 0.324) and FI (SMD: −0.085, 95% CI: −0.311, 0.141) without significant difference. No result was presented in the subgroup analysis of WP with RT as there was only one study available for each outcome. (Figures S13–S16)

In the subgroup without RT, WP supplementation may enhance CHI and reduce PI, FI, and TEI. A significant effect was demonstrated in PI (SMD: −0.4225, 95% CI: −0.774, −0.071), while that of CHI (SMD: 0.038, 95% CI: −0.356, 0.432), FI (SMD: −0.1135, 95% CI: −0.352, 0.125), and TEI (SMD: −0.1225, 95% CI: −0.361, 0.116) were not significant.

### 3.11. Quality of Evidence Assessment by GRADE

The results of the GRADE evaluation are presented in Table 3. We evaluated all of the outcomes with more than one paper providing information. Therefore, all 17 outcomes were assessed in the analysis of combining all included studies, while only 11 and 10 outcomes were assessed in the subgroup analysis of WP supplementation with and without RT, respectively. As the design of the inclusion criteria was rigid, there was no obvious intransitivity. In the analysis of combining all of the included studies, the confidence of the evidence of FI and TEI were high for no downgrading, while that of FM was very low due to the inconsistency, imprecision, and some concerns in relation to the risk of bias. In the subgroup assessment, the extent of inconsistency decreased, while that of imprecision increased. For the subgroup of WP supplementation with RT, the confidence of the evidence of KE and KF was very low due to the imprecision and high risk in relation to the risk of bias. For the subgroup analysis of WP supplementation without RT, the confidence of the evidence of FM was very low due to the inconsistency, imprecision, and some concerns in relation to the risk of bias.

## 4. Discussion

Based on evidence, this review summarized the effect of WP supplementation on postmenopausal women. The main findings of this meta-analysis are: (1) WP with RT significantly enhances LLLM gain and BC; (2) WP without RT significantly reduces PI.

### 4.1. Effect of WP Supplementation on Muscle Strength and Functional Capacity

Subgroup analysis showed that RT augments the benefits of whey protein for muscle strength. WP supplementation in the group with RT demonstrated a significant enhancement of BC, while that without RT showed no significant enhancement of all kinds of muscle strength and functional capacity. The analysis results partially agree with previous meta-analyses by Finger et al., Morton et al., and Liao et al. [19,20,37], which investigated the effect of protein supplementation together with RT, and a previous meta-analysis by Richard et al., suggesting that protein interventions augment the effect of RT on muscle strength in older adults. Therefore, despite the difference in metabolism between men and women, for postmenopausal women consuming sufficient quantities of protein, WP supplementation still enhances muscle strength, but only when combined with RT. A possible explanation is that RT increases fasted-state protein losses and the need for protein. In this condition, WP supplementation promotes maintaining whole body protein-balance [38].

Another study concluded that the improvement in strength was related to better physical and social function [39]. Nevertheless, in our study, no significant effect on functional capacity was revealed. More studies with uniformity in outcome measures would be needed to clarify this conclusion.

### 4.2. Effect of WP Supplementation on Muscle Mass

RT played an important role for muscle mass in the subgroup analysis. WP supplementation in the RT group showed a significant enhancement in LLLM. However, the group without RT demonstrated a significant decrease in ULLM and LLLM. The results of our analysis partially agree with previous meta-analyses [19,20,21,37], suggesting that protein interventions augment the effect of RT on appendicular lean mass. For the significant decrease in ULLM and LLLM, the results were constructed by only one study and the intervention time of this study was one year [34], while that of the studies presenting the result of WP with RT was 10–24 weeks, and the effect of age-related decline was taken into consideration. Moreover, this study used an isocaloric carbohydrate for supplementation in control group. Carbohydrate stimulates pancreatic insulin secretion, which can inhibit muscle protein breakdown [40,41]. Elderly people of normal status have lower insulin secretion [42]. This might explain the decrease in ULLM and LLLM.

The results of WP supplementation in the RT group did not show a significant effect on lower limb muscle strength, but demonstrated a significant enhancement of LLLM. A previous study presented the same outcome [43], showing that the relationship between muscle strength and muscle mass differed according to sex and age. For women between 65 and 74 years, no significant relationship between muscle strength and muscle mass was demonstrated. Both “neural” and “muscular” factors are required for muscle strength; meanwhile, this neural activity decreases with aging [44]. In addition, strength tests are performance tests and affected by technique and motivation. In novice individuals, there is more testing variability. These might explain the lack of a relationship between lower limb muscle strength and LLLM.

### 4.3. Effect of WP Supplementation on FM and BW Loss

Subgroup analysis showed that RT was not a key factor in FM and BW loss. Both WP groups, with and without RT, showed a positive effect on FM loss, corresponding to most studies showing that increasing dietary protein increases diet-induced thermogenesis and promotes greater fat loss. Compared to the group of WP without RT, WP supplementation with RT showed less effect on FM loss. A possible explanation is that the intervention time of this study was 18 months [33], while that of the other three studies which provided the results of FM loss were 12–16 weeks [23,24,27]. With a longer intervention time, the effect on FM loss might be more significant.

Our results demonstrated that WP supplementation might not be necessary to be combined with RT for FM loss in postmenopausal women.

### 4.4. Effect of WP Supplementation on Daily Dietary Nutrients Intake

WP supplementation in the group without RT demonstrated a negative effect on PI without significant difference, while that with RT showed a significant positive effect on PI. The results of our analysis partially agree with a previous meta-analysis by Colonetti et al. [45], which investigated the effect of dietary protein supplementation together with RT. For the negative effect on PI in the group without RT, it might be explained by the use of the dietary nutrient intake with deduction of WP and placebo supplementation in our study. The Study Group on meeting protein needs of older people (PROT-AGE) and the European Society for Clinical Nutrition and Metabolism (ESPEN) study showed that 1.0–1.2 g/kg BM^−1^ day^−1^ of protein for well-nourished active older adults is sufficient [46,47], while RT can increase fasted-state protein losses and the need of protein. Total protein intake remained unchanged in the group of WP without RT. With extra supplementation of protein, a new balance of dietary intake was achieved, thus contributing to a decline in PI. This might indirectly conclude that WP supplementation was not very helpful for postmenopausal women without RT, especially for those who already consumed sufficient quantities of protein at baseline. On the contrary, for those who underwent RT, WP supplementation might augment the effect of RT and increase the need for protein. Therefore, WP supplementation should be combined with RT for increasing daily dietary PI in postmenopausal women.

### 4.5. Limitation

First, due to restricted number of included studies, subgroup analysis other than RT/non-RT is not constructed in our study. We did not divide the group into trained/untrained individuals, healthy/sarcopenia individuals, sufficient/deficient PI individuals, or build a subgroup analysis of protein supplementation dosage. We also did not discriminate the role of WP from being a supplement or a correction of a deficient diet. Furthermore, some studies suggested that resistance-trained individuals might need a higher dietary intake and protein supplementation [20,41]. Second, the intervention time in non-RT studies is longer than RT studies. The adherence to RT, WP, and PLA supplementation, and the effect of aging on muscle mass, muscle strength, and FM should be considered. Third, we included lean mass, muscle mass, and lean soft tissue mass in the analysis of lean mass gain, and the variation in bone and water should be also considered. Fourth, participants in Sugihara et al. had a basal PI lower than the current adult-recommended dietary allowances, and the results of TEI, CHI, PI, and FI should be interpreted with caution. Last, the restricted number of included studies might lead to some results not showing a significant effect.

## 5. Conclusions

Compared to placebo control, WP supplementation causes an improvement in BC and LLLM in postmenopausal women, only when combined with RT. However, the quality of evidence of BC and LLLM in the group of WP with RT was low and moderate, respectively. With both of them presenting some risk of bias, the results should be interpreted with caution. More large scale RCTs are required for a better understanding of the effects of WP supplementation combined with RT.

## Figures and Tables

**Table 1:** Characteristics of the intervention studies.

Study, Year Country	Intervention Duration	Number of Female Participants	Female Participant Health Status	Resistance Training	Whey Protein Supplementation	Placebo Supplementation	Outcome Measured and Used
Ashley et al., 2021 [25] USA	12 weeks	With RT: 67	Well-fed healthy	3 days/week 3 sets × 8–12 reps 2 upper limb exercises 1 lower limb exercise 1 aerobic exercise	20 g × 2/day post RT or before meal	maltodextrin non-isocaloric non-nitrogenous	CP GST GS BW FM
Kirk et al., 2020 [23] UK	16 weeks	With RT: 25 Without RT: 27	Non-frail healthy RT naïve	2 days/week 2 sets to fatigue 4 upper limb exercises 3 lower limb exercises moderate weight	1.5 g/kg BM^−1^ day^−1^	-	SMM KF GS KE FM
Nabuco et al., 2019, 1st [26] Brazil	12 weeks	With RT: 44	Healthy or sarcopenic RT naïve	3 days/week 3 sets × 8–12 reps 4 upper limb exercises 4 lower limb exercises	27.1 g post RT	maltodextrin isocaloric nitrogenous	-
Nabuco et al., 2019, 2nd [27] Brazil	12 weeks	With RT: 44	Healthy or sarcopenic RT naïve	3 days/week 3 sets × 8–12 reps 4 upper limb exercises 4 lower limb exercises	27.1 g post RT	maltodextrin isocaloric nitrogenous	FM
Nabuco et al., 2019, 3rd [28] Brazil	12 weeks	With RT: 30	Healthy or sarcopenic RT naïve	3 days/week 3 sets × 8–12 reps 4 upper limb exercises 4 lower limb exercises	27.1 g post RT	maltodextrin isocaloric nitrogenous	-
Nabuco et al., 2019, 4th [29] Brazil	12 weeks	With RT: 26	Sarcopenic RT naïve	3 days/week 3 sets × 8–12 reps 4 upper limb exercises 4 lower limb exercises	27.1 g post RT	maltodextrin isocaloric nitrogenous	-
Sugihara et al., 2018 [30] Brazil	12 weeks	With RT: 31	Healthy or sarcopenic RT naïve	3 days/week 3 sets × 8–12 reps 4 upper limb exercises 4 lower limb exercises	27.1 g post RT	maltodextrin isocaloric nitrogenous	TEI CHI PI FI
Nabuco et al., 2018 [31] Brazil	12 weeks	With RT: 44	Healthy or sarcopenic RT naïve	3 days/week 3 sets × 8–12 reps 4 upper limb exercises 4 lower limb exercises	27.1 g post RT	maltodextrin isocaloric nitrogenous	ULLM SMM BC CP GST LLLM KE RFSP
Mori et al., 2018 [24] Japan	24 weeks	With RT: 50	Healthy RT naïve	2 days/week >40 min 2 upper limb exercises 5 lower limb exercises 50–70% 1RM	22.3 g post RT	-	ULLM GST LLLM GS KE BW
Fernandes et al., 2018 [32] Brazil	12 weeks	With RT: 32	Healthy or sarcopenic RT naïve	3 days/week 3 sets × 8–12 reps 4 upper limb exercises 4 lower limb exercises	27.1 g post RT	maltodextrin isocaloric nitrogenous	-
Stojkovic et al., 2017 [33] USA	72 weeks	Without RT: 84	-	-	20 g	maltodextrin isocaloric	FM TEI CHI PI FI
Zhu et al., 2015 [34] Australia	52 weeks	Without RT: 196	-	-	30 g (milk and whey protein)	carbohydrate isocaloric nitrogenous	ULLM SMM KF LLLM GS KE BW RFSP
Weisgarber et al., 2014 [35] Canada	10 weeks	With RT: 12	Healthy RT naïve	2 days/week 0–6 sets to fatigue 2 upper limb exercises 2 lower limb exercises 30% 1 RM	40 g	maltodextrin isocaloric non-nitrogenous	BC KF KE
Hodgson et al., 2012 [36] Australia	52 weeks	Without RT: 196	-	-	30 g (milk and whey protein)	carbohydrate isocaloric nitrogenous	TEI CHI PI FI

BC = biceps curl strength; BW = body weight; CHI = carbohydrate intake; CP = chest press; FI = fat intake; FM = fat mass; GS = grip strength; GST = gait speed test; KE = knee extension strength; KF = knee flexion strength; LLLM = lower limb lean mass; PI = protein intake; PLA = placebo; reps = repetitions; RT = resistance training; RFSP = rising from sitting position; SMM = skeletal muscle mass; TEI = total energy intake; ULLM = upper limb lean mass; WP = whey protein.

**Table 2:** Participant characteristics of the intervention studies.

Study, Year	Age	BMI (kg·m^−2^)	FM (kg)	KE (kg)	GS (kg)	Relative Protein Intake (g/kg BM^−1^ Day^−1^)
Ashley et al., 2021 [25]	61.93 ± 1.23 (WP) 60.64 ± 0.93 (PLA)	-	23.639 ± 1.37 (WP) 24.25 ± 0.89 (PLA)	-	26.851 ± 0.80 (WP) 27.88 ± 0.75 (PLA)	1.1 ± 0.09 (WP) 1.20 ± 0.09 (PLA)
Nabuco et al., 2018, 2019 1st, 2nd [26,27,31]	66.2 ± 9.4 (WP) 66.5 ± 7.1 (PLA)	25.3 ± 5.4 (WP) 23.8 ± 3.7 (PLA)	23.2 ± 8.4 (WP) 22.9 ± 7.5 (PLA)	55.0 ± 11.0 (WP) 52.0 ± 13.0 (PLA)	-	0.94 ± 0.34 (WP) 0.95 ± 0.27 (PLA)
Nabuco et al., 2019, 4th [29]	68.0 ± 4.2 (WP) 70.1 ± 3.9 (PLA)	26.4 ± 3.0 (WP) 27.4 ± 3.0 (PLA)	23.8 ± 5.4 (WP) 23.8 ± 5.9 (PLA)	48.7 ± 10.8 (WP) 50.9 ± 9.9 (PLA)	-	0.93 ± 0.36 (WP) 0.97 ± 0.28 (PLA)
Mori et al., 2018 [24]	70.6 ± 4.2 (WP) 70.6 ± 4.2 (control)	22.1 ± 2.1 (WP) 22.9 ± 2.9 (control)	-	23.8 ± 6.3 (WP) 26.7 ± 3.8 (control)	22.4 ± 3.4 (WP) 23.1 ± 5.3 (control)	1.3 ± 0.0 (WP) 1.3 ± 0.0 (control)
Stojkovic et al., 2017 [33]	68.9 ± 0.9 (WP) 69.3 ± 0.9 (PLA)	26.0 ± 0.6 (WP) 25.8 ± 0.6 (PLA)	25.9 ± 1.3 (WP) 25.5 ± 1.1 (PLA)	-	-	-
Weisgarber et al., 2014 [35]	57 ± 4.7	28.3 ± 7.0	-	59.7 ± 15.3 (WP) 61.0 ± 16.1 (PLA)	-	-
Kirk et al., 2020 (RT) [23]	69 ± 6 (WP) 66 ± 4 (control)	27.4 ± 4.9 (WP) 28.1 ± 7.4 (control)	22.8 ± 10.5 (WP) 28.2 ± 17.6 (control)	173 ± 46 (MVC) (WP) 233 ± 126 (MVC) (control)	23.2 ± 5.5 (MVC) (WP) 21.7 ± 4.8 (MVC) (control)	1.16 ± 0.4 (WP) 1.10 ± 0.4 (control)
Kirk et al., 2020 (non-RT) [23]	72 ± 6 (WP) 68 ± 6 (control)	27.1 ± 4.1 (WP) 26.2 ± 4.5 (control)	27.6 ± 8.5 (WP) 25.5 ± 11.9 (control)	190 ± 105 (MVC) (WP) 180 ± 49 (MVC) (control)	22.4 ± 4.4 (MVC) (WP) 23.9 ± 4.1 (MVC) (control)	0.99 ± 0.2 (WP) 0.98 ± 0.3 (control)
Nabuco et al., 2019, 3rd [28]	69.2 ± 4.1 (WP) 68.4 ± 4.5 (PLA)	27.4 ± 5.1 (WP) 26.6 ± 3.4 (PLA)	-	-	-	0.94 ± 0.30 (WP) 0.96 ± 0.22 (PLA)
Sugihara et al., 2018 [30]	67.4 ± 4.1 (WP) 67.8 ± 4.1 (PLA)	25.6 ± 2.4 (WP) 25.4 ± 2.6 (PLA)	25.7 ± 4.6 (WP) 26.2 ± 5.8 (PLA)	52.7 ± 10.3 (WP) 52.8 ± 13.3 (PLA)	-	0.85 ± 0.1 (WP) 0.81 ± 0.1 (PLA)
Fernandes et al., 2018 [32]	67.3 ± 4.1 (WP) 67.8 ± 4.0 (PLA)	25.9 ± 2.7 (WP) 25.4 ± 2.6 (PLA)	25.7 ± 4.6 (WP) 26.2 ± 5.8 (PLA)	52.7 ± 10.3 (WP) 52.8 ± 13.3 (PLA)	-	0.85 ± 0.1 (WP) 0.81 ± 0.1 (PLA)
Zhu et al., 2015 [34]	74.2 ± 2.8 (WP) 74.3 ± 2.6 (PLA)	26.1 ± 3.8 (WP) 27.2 ± 4.0 (PLA)	-	15.4 ± 5.3 (WP) 16.1 ± 7.2 (PLA)	21.7 ± 5.2 (WP) 21.7 ± 5.5 (PLA)	1.2 ± 0.3 (WP) 1.1 ± 0.3 (PLA)
Hodgson et al., 2012 [36]	74.2 ± 2.8 (WP) 74.3 ± 2.6 (PLA)	26.1 ± 3.8 (WP) 27.2 ± 4.0 (PLA)	-	-	-	1.1 ± 0.3 (WP) 1.1 ± 0.3 (PLA)

FM = fat mass; KE = knee extension strength; GS = grip strength; MVC= maximum voluntary contraction; PLA = placebo; RT = resistance training; WP = whey protein.

**Table 3:** The results and GRADE evaluation of all outcomes.

Combined WP with RT and without RT								
Outcome (PLA vs. WP)	Number of Articles	Mean Difference, with 95% Confidence Interval	*I* ^2^	Risk of Bias	Inconsistency	Indirectness	Imprecision	Quality of Evidence
Upper limb lean mass	3	−0.2415 (−0.473, −0.01)	1.5%	−1	−0	−0	−0	Moderate
Lower limb lean mass	3	0.028 (−0.214, 0.27)	93.6%	−1	−1	−0	−0	Low
Skeletal muscle mass	3	0.08 (−0.533, 0.693)	67%	−1	−1	−0	−0	Low
Grip strength	5	0.1005 (−0.107, 0.308)	0%	−1	−0	−0	−0	Moderate
Biceps curl strength	2	0.6805 (0.176, 1.185)	0%	−2	−0	−0	−0	Low
Knee extension strength	6	0.027 (−0.194, 0.248)	44%	−2	−0	−0	−0	Low
Knee flexion strength	4	0.044 (−0.214, 0.302)	22%	−2	−0	−0	−0	Low
Gait speed test	3	0.1625 (−0.261, 0.586)	44%	−1	−0	−0	−1	Low
Rising from sitting position	2	−0.036 (−0.291, 0.219)	0%	−1	−0	−0	−0	Moderate
Chest press	2	0.108 (−0.268, 0.484)	0%	−1	−0	−0	−1	Low
Fat mass	5	−0.107 (−0.443, 0.229)	93%	−1	−1	−0	−1	Very low
Body weight	4	−0.115 (−0.291, 0.061)	0%	−1	−0	−0	−0	Moderate
Protein intake	3	−0.0685 (−0.396, 0.259)	94%	−0	−1	−0	−1	Low
Fat intake	3	−0.085 (−0.311, 0.141)	0%	−0	−0	−0	−0	High
Carbohydrate intake	3	−0.0205 (−0.365, 0.324)	32%	−0	−0	−0	−1	Moderate
Total energy intake	3	−0.067 (−0.293, 0.159)	2.84%	−0	−0	−0	−0	High
**WP with RT**								
Upper limb lean mass	2	0 (−0.405, 0.405)	0%	−1	−0	−0	−1	Low
Lower limb lean mass	2	1.103 (0.632, 1.574)	14%	−1	−0	−0	−0	Moderate
Skeletal muscle mass	2	0.4775 (−0.473, 1.428)	72%	−1	−0	−0	−1	Low
Grip strength	3	0.1765 (−0.156, 0.509)	0%	−1	−0	−0	−0	Moderate
Biceps curl strength	2	0.6805 (0.176, 1.185)	0%	−2	−0	−0	−0	Low
Knee extension strength	4	0.364 (−0.031, 0.759)	24%	−2	−0	−0	−1	Very low
Knee flexion strength	2	0.2905 (−0.756, 1.337)	67%	−2	−1	−0	−1	Very low
Gait speed test	3	0.1625 (−0.261, 0.586)	44%	−1	−0	−0	−1	Low
Chest press	2	0.108 (−0.268, 0.484)	0%	−1	−0	−0	−1	Low
Fat mass	3	−0.0885 (−0.427, 0.25)	0%	−1	−0	−0	−1	Low
Body weight	2	−0.0205 (−0.386, 0.345)	0%	−1	−0	−0	−1	Low
**WP without RT**								
Grip strength	2	0.0515 (−0.215, 0.318)	0%	−1	−0	−0	−0	Moderate
Knee extension strength	2	−0.1255 (−0.392, 0.141)	0%	−1	−0	−0	−0	Moderate
Knee flexion strength	2	0.0285 (−0.238, 0.295)	0%	−1	−0	−0	−0	Moderate
Fat mass	2	−1.2825 (−3.975, 1.410)	96%	−1	−1	−0	−1	Very low
Body weight	2	−0.1435 (−0.344, 0.057)	0%	−1	−0	−0	−0	Moderate
Protein intake	2	−0.4225 (−0.774, −0.071)	47%	−0	−1	−0	−0	Moderate
Fat intake	2	−0.1135 (−0.352, 0.125)	0%	−0	−0	−0	−0	High
Carbohydrate intake	2	0.038 (−0.356, 0.432)	58%	−0	−0	−0	−1	Moderate
Total energy intake	2	−0.1225 (−0.361, 0.116)	0%	−0	−0	−0	−0	High

PLA = placebo; RT = resistance training; WP = whey protein.

## Data Availability

Data were available within the article and its supplementary materials.

## Supplementary Materials

The following supporting information can be downloaded at: https://www.mdpi.com/article/10.3390/nu14194210/s1, Method S1: The full search strings: example of PubMed; Table S1: PRISMA 2020 checklist; Table S2: Differences in muscle strength between whey protein supplements and control supplements considering the subgroup analysis of RT/non-RT; Table S3: Differences in muscle mass between whey protein supplements and control supplements considering the subgroup analysis of RT/non-RT; Table S4: Differences in fat mass and body weight between whey protein supplements and control supplements considering the subgroup analysis of RT/non-RT; Table S5: Differences in daily nutrients intake between whey protein supplements and control supplements considering the subgroup analysis of RT/non-RT; Figure S1: Forest plot of the grip strength; Figure S2: Forest plot of the biceps curl strength; Figure S3: Forest plot of the knee extension strength; Figure S4: Forest plot of the knee flexion strength; Figure S5: Forest plot of the gait speed test; Figure S6: Forest plot of the chest press; Figure S7: Forest plot of the rising from sitting position; Figure S8: Forest plot of the upper limb lean mass; Figure S9: Forest plot of the lower limb lean mass; Figure S10: Forest plot of the skeletal muscle mass; Figure S11: Forest plot of the fat mass; Figure S12: Forest plot of the body weight; Figure S13: Forest plot of the total energy intake; Figure S14: Forest plot of the carbohydrate intake; Figure S15: Forest plot of the fat intake; Figure S16: Forest plot of the protein intake.

## Abbreviation

BC	Biceps curl strength
BW	Body weight
CHI	Carbohydrate intake
CIs	Confidence intervals
CP	Chest press
FI	Fat intake
FM	Fat mass
GS	Grip strength
GST	Gait speed test
KE	Knee extension strength
KF	Knee flexion strength
LLLM	Lower limb lean mass
PI	Protein intake
PLA	Placebo
RCTs	Randomized controlled trials
reps	Repetitions
RT	Resistance training
RFSP	Rising from sitting position
SMM	Skeletal muscle mass
SMDs	Standardized mean differences
TEI	Total energy intake
GRADE	The Grading of Recommendations, Assessment, Development, and Evaluations
ULLM	Upper limb lean mass
WP	Whey protein
