# Supplementary material for: Effect of Whey Protein Supplementation in Postmenopausal Women: A Systematic Review and Meta-Analysis
Source: Nutrients. 2022 Oct 10;14(19):4210. doi: 10.3390/nu14194210 (PMC9572824; doi:10.3390/nu14194210)

Supporting information

Supplemental Method S1. The full search strings: example of PubMed

Supplemental Table S1. PRISMA 2020 checklist

Supplemental Table S2. Differences in muscle strength between whey protein supplements and control supplements considering the subgroup analysis of RT/non-RT

Supplemental Table S3. Differences in muscle mass between whey protein supplements and control supplements considering the subgroup analysis of RT/non-RT

Supplemental Table S4. Differences in fat mass and body weight between whey protein supplements and control supplements considering the subgroup analysis of RT/non-RT

Supplemental Table S5. Differences in daily nutrients intake between whey protein supplements and control supplements considering the subgroup analysis of RT/non-RT

Supplemental Figure S1. Forest plot of the grip strength

Supplemental Figure S2. Forest plot of the biceps curl strength

Supplemental Figure S3. Forest plot of the knee extension strength

Supplemental Figure S4. Forest plot of the knee flexion strength

Supplemental Figure S5. Forest plot of the gait speed

Supplemental Figure S6. Forest plot of the chest press

Supplemental Figure S7. Forest plot of the rising from sitting position

Supplemental Figure S8. Forest plot of the upper limb lean mass

Supplemental Figure S9. Forest plot of the lower limb lean mass

Supplemental Figure S10. Forest plot of the skeletal muscle mass

Supplemental Figure S11. Forest plot of the fat mass

Supplemental Figure S12. Forest plot of the body weight

Supplemental Figure S13. Forest plot of the total energy intake

Supplemental Figure S14. Forest plot of the carbohydrate intake

Supplemental Figure S15. Forest plot of the fat intake

Supplemental Figure S16. Forest plot of the protein intake

Supplemental Method S1. The full search strings: example of PubMed

Search terms	Number
#1 "whey protein "[MeSH Terms] OR " whey protein "[All Fields]	4610
#2 (" menopause women "[MeSH Terms] OR " menopause women "[All Fields]) OR ("older women "[MeSH Terms] OR " older women "[All Fields])	16574
#3 #1 AND #2	23

Supplemental Table S1. PRISMA 2020 checklist

Section and Topic	Item #	Checklist item	Location where item is reported
**TITLE**			
Title	1	Identify the report as a systematic review.	1
**ABSTRACT**			
Abstract	2	See the PRISMA 2020 for Abstracts checklist.	1
**INTRODUCTION**			
Rationale	3	Describe the rationale for the review in the context of existing knowledge.	1-2
Objectives	4	Provide an explicit statement of the objective(s) or question(s) the review addresses.	2
**METHODS**			
Eligibility criteria	5	Specify the inclusion and exclusion criteria for the review and how studies were grouped for the syntheses.	3
Information sources	6	Specify all databases, registers, websites, organizations, reference lists and other sources searched or consulted to identify studies. Specify the date when each source was last searched or consulted.	2-3
Search strategy	7	Present the full search strategies for all databases, registers, and websites, including any filters and limits used.	2-3 Supplemental Method 1
Selection process	8	Specify the methods used to decide whether a study met the inclusion criteria of the review, including how many reviewers screened each record and each report retrieved, whether they worked independently, and if applicable, details of automation tools used in the process.	3

Section and Topic	Item #	Checklist item	Location where item is reported
Data collection process	9	Specify the methods used to collect data from reports, including how many reviewers collected data from each report, whether they worked independently, any processes for obtaining or confirming data from study investigators, and if applicable, details of automation tools used in the process.	3
Data items	10a	List and define all outcomes for which data were sought. Specify whether all results that were compatible with each outcome domain in each study were sought (e.g. for all measures, time points, analyses), and if not, the methods used to decide which results to collect.	3
	10b	List and define all other variables for which data were sought (e.g. participant and intervention characteristics, funding sources). Describe any assumptions made about any missing or unclear information.	3
Study risk of bias assessment	11	Specify the methods used to assess risk of bias in the included studies, including details of the tool(s) used, how many reviewers assessed each study and whether they worked independently, and if applicable, details of automation tools used in the process.	3
Effect measures	12	Specify for each outcome the effect measure(s) (e.g. risk ratio, mean difference) used in the synthesis or presentation of results.	3
Synthesis methods	13a	Describe the processes used to decide which studies were eligible for each synthesis (e.g. tabulating the study intervention characteristics and comparing against the planned groups for each synthesis (item #5)).	3
	13b	Describe any methods required to prepare the data for presentation or synthesis, such as handling of missing summary statistics, or data conversions.	3
	13c	Describe any methods used to tabulate or visually display results of individual studies and syntheses.	3
	13d	Describe any methods used to synthesize results and provide a rationale for the choice(s). If meta-analysis was performed, describe the model(s), method(s) to identify the presence and extent of statistical heterogeneity, and software package(s) used.	3
	13e	Describe any methods used to explore possible causes of heterogeneity among study results (e.g. subgroup analysis, meta-regression).	3

Section and Topic	Item #	Checklist item	Location where item is reported
	13f	Describe any sensitivity analyses conducted to assess robustness of the synthesized results.	3
Reporting bias assessment	14	Describe any methods used to assess risk of bias due to missing results in a synthesis (arising from reporting biases).	3
Certainty assessment	15	Describe any methods used to assess certainty (or confidence) in the body of evidence for an outcome.	3
**RESULTS**			
Study selection	16a	Describe the results of the search and selection process, from the number of records identified in the search to the number of studies included in the review, ideally using a flow diagram.	4, Figure 1
	16b	Cite studies that might appear to meet the inclusion criteria, but which were excluded, and explain why they were excluded.	4-5
Study characteristics	17	Cite each included study and present its characteristics.	4-6, Table 1
Risk of bias in studies	18	Present assessments of risk of bias for each included study.	7, Figure 2
Results of individual studies	19	For all outcomes, present, for each study: (a) summary statistics for each group (where appropriate) and (b) an effect estimate and its precision (e.g. confidence/credible interval), ideally using structured tables or plots.	8-9, Supplemental Tables 2–5
Results of syntheses	20a	For each synthesis, briefly summaries the characteristics and risk of bias among contributing studies.	7-10
	20b	Present results of all statistical syntheses conducted. If meta-analysis was done, present for each the summary estimate and its precision (e.g.	7-10,

Section and Topic	Item #	Checklist item	Location where item is reported
		confidence/credible interval) and measures of statistical heterogeneity. If comparing groups, describe the direction of the effect.	Supplemental Figure 1-16, Table 2
	20c	Present results of all investigations of possible causes of heterogeneity among study results.	N/A
	20d	Present results of all sensitivity analyses conducted to assess the robustness of the synthesized results.	N/A
Reporting biases	21	Present assessments of risk of bias due to missing results (arising from reporting biases) for each synthesis assessed.	N/A
Certainty of evidence	22	Present assessments of certainty (or confidence) in the body of evidence for each outcome assessed.	10, Table 3
**DISCUSSION**			
Discussion	23a	Provide a general interpretation of the results in the context of other evidence.	10-12
	23b	Discuss any limitations of the evidence included in the review.	12
	23c	Discuss any limitations of the review processes used.	12
	23d	Discuss implications of the results for practice, policy, and future research.	12
**OTHER INFORMATION**			
Registration and protocol	24a	Provide registration information for the review, including register name and registration number, or state that the review was not registered.	2
	24b	Indicate where the review protocol can be accessed, or state that a protocol was not prepared.	2
	24c	Describe and explain any amendments to information provided at registration or in the protocol.	N/A
Support	25	Describe sources of financial or non-financial support for the review, and the role of the funders or sponsors in the review.	N/A

Section and Topic	Item #	Checklist item	Location where item is reported
Competing interests	26	Declare any competing interests of review authors.	N/A
Availability of data, code and other materials	27	Report which of the following are publicly available and where they can be found: template data collection forms; data extracted from included studies; data used for all analyses; analytic code; any other materials used in the review.	N/A

Supplemental Table S2. Differences in muscle strength between whey protein supplements and control supplements considering the subgroup analysis of RT/non-RT

Grip strength (kg)

	Whey protein			Control			Std diff in
**Study**	**Mean**	**SD**	**Total**	**Mean**	**SD**	**Total**	**means and 95% CI**
**With RT**							
Mori 2018 [24]	1.2	1.45	25	0.6	1.74	25	0.3745 [-0.185, 0.934]
Kirk 2020 [23]	3.8	8.02	13	2.7	4.3	12	0.169 [-0.617, 0.955]
Ashley 2021 [25]	2.249	4.95	28	2.11	4.48	39	0.0295 [-0.456, 0.515]
**Without RT**							
Kirk 2020 [23]	0.4	4.45	9	0.5	4.26	18	-0.023 [-0.823, 0.777]
Zhu 2015 [34]	-0.87	3.98	99	-1.11	3.88	94	0.061 [-0.221, 0.343]

Knee flexion strength (kg)

	Whey protein			Control			Std diff in
**Study**	**Mean**	**SD**	**Total**	**Mean**	**SD**	**Total**	**means and 95% CI**
**With RT**							
Weisgarber 2014 [35]	-1.7	10.41	10	1	10.61	10	-0.257 [-1.137, 0.623]
Kirk 2020 [23]	52	39.2	13	-1	85.1	12	0.8115 [-0.005, 1.628]
**Without RT**							
Kirk 2020 [23]	-9	128	9	-3	44.2	18	-0.074 [-0.874, 0.726]
Zhu 2015 [34]	1.81	3.78	99	1.65	3.98	94	0.0415 [-0.241, 0.324]

Supplemental Table S2. Cont.

Chess press (kg)

Study	Whey protein Mean	Whey protein SD	Whey protein Total	Control Mean	Control SD	Control Total	Std diff in means and 95% CI
**With RT**							
Nabuco 2018 [31]	3	9.54	21	2	8	23	0.114 [-0.478, 0.706]
Ashley 2021 [25]	5.106	5.298	28	4.57	5.05	39	0.104 [-0.382, 0.590]

Gait speed test (m/s)

Study	Whey protein Mean	Whey protein SD	Whey protein Total	Control Mean	Control SD	Control Total	Std diff in means and 95% CI
**With RT**							
Mori 2018 [24]	0	0.1	25	0	0.173	25	0 [-0.554, 0.554]
Nabuco 2018 [31]	0.194	0.213	21	0.0735	0.161	23	0.6425 [0.036, 1.249]
Ashley 2021 [25]	1.575	3.64	28	1.8037	2.83	39	-0.0715 [-0.557, 0.414]

Biceps curl strength (kg)

Study	Whey protein Mean	Whey protein SD	Whey protein Total	Control Mean	Control SD	Control Total	Std diff in means and 95% CI
**With RT**							
Weisgarber 2014 [35]	5.8	4.55	10	3.5	4.26	10	0.522 [-0.369, 1.413]
Nabuco 2018 [31]	3	1.6	21	2	1.01	23	0.755 [0.143, 1.367]

Supplemental Table S2. Cont.

Knee extension strength (kg)

Study	Whey protein Mean	SD	Total	Control Mean	SD	Total	Std diff in means and 95% CI
**With RT**							
Weisgarber 2014 [35]	5.5	13.44	10	5.2	14.97	10	0.0215 [-0.855, 0.898]
Nabuco 2018 [31]	4	1	21	4	0.53	23	0 [-0.592, 0.592]
Mori 2018 [24]	2.6	2.85	25	1.1	1.625	25	0.1465 [0.078, 0.215]
Kirk 2020 [23]	106	78.8	13	33	109	12	0.773 [-0.04, 1.586]
**Without RT**							
Kirk 2020 [23]	-17	91	9	-9	46.7	18	-0.1245 [-0.925, 0.676]
Zhu 2015 [34]	2.08	5.37	99	2.83	6.5	94	-0.1265 [-0.409, 0.156]

Rising from sitting position (s)

Study	Whey protein Mean	SD	Total	Control Mean	SD	Total	Std diff in means and 95% CI
**With RT**							
Nabuco 2018 [31]	-1.2	1.61	21	-0.7	1.8	23	-0.292 [-0.887, 0.303]
**Without RT**							
Zhu 2015 [23]	-0.14	1.29	99	-0.17	1.454	94	0.022 [-0.260, 0.304]

RT = resistance training; SD = standard deviation; Std diff = standard difference; CI = confidence interval

Supplemental Table S3. Differences in muscle mass between whey protein supplements and control supplements considering the subgroup analysis of RT/non-RT

Upper limb lean mass (kg)

	Whey protein			Control			Std diff in
**Study**	**Mean**	**SD**	**Total**	**Mean**	**SD**	**Total**	**means and 95% CI**
**With RT**							
Mori 2018 [24]	0	0.0242	25	0	0.0181	25	0 [-0.554, 0.554]
Nabuco 2018 [31]	0.2	0.146	21	0.2	0.171	23	0 [-0.592, 0.592]
**Without RT**							
Zhu 2015 [34]	0.02	0.2	101	0.09	0.19	95	0.3585 [-0.641, -0.076]

Lower limb lean mass (kg)

	Whey protein			Control			Std diff in
**Study**	**Mean**	**SD**	**Total**	**Mean**	**SD**	**Total**	**means and 95% CI**
**With RT**							
Mori 2018 [24]	0.5	0.25	25	0.3	0.198	25	0.887 [0.306, 1.468]
Nabuco 2018 [31]	0.4	0.238	21	0.1	0.2	23	1.3705 [0.713, 2.028]
**Without RT**							
Zhu 2015 [34]	0.02	0.2	101	0.09	0.19	95	-0.3585 [-0.641, -0.076]

Supplemental Table S3. Cont.

Skeletal muscle mass (kg)							
		Whey protein			Control		Std diff in
Study	Mean	SD	Total	Mean	SD	Total	means and 95% CI
**With RT**							
Nabuco 2018 [31]	0.8	0.438	21	0.4	0.42	23	0.933 [0.31, 1.556]
Kirk 2020 [23]	0.2	2.75	13	0.3	2.33	12	-0.039 [-0.824, 0.746]
**Without RT**							
Kirk 2020 [23]	-0.3	2.62	9	0.2	2.4	18	-0.202 [-1.004, 0.600]

RT = resistance training; SD = standard deviation; Std diff = standard difference; CI = confidence interval

Supplemental Table S4. Differences in fat mass and body weight between whey protein supplements and control supplements considering the subgroup analysis of RT/non-RT

Fat mass (kg)

	Whey protein			Control			Std diff in
**Study**	**Mean**	**SD**	**Total**	**Mean**	**SD**	**Total**	**means and 95% CI**
**With RT**							
Kirk 2020 [23]	-0.2	10.35	13	-0.5	16.81	12	0.0215 [-0.763, 0.806]
Nabuco 2019 [27]	-0.6	8.35	21	-0.1	7.55	23	-0.063 [-0.655, 0.529]
Ashley 2021 [25]	-0.319	7.17	28	0.76	7.375	39	-0.148 [-0.634, 0.338]
**Without RT**							
Kirk 2020 [23]	1	9.4	9	-0.2	12.21	18	0.1055 [-0.695, 0.906]
Stojkovic 2017 [33]	-0.009	0.4	38	1.2	0.5	46	-2.642 [-3.229, -2.055]

Body weight (kg)

	Whey protein			Control			Std diff in
**Study**	**Mean**	**SD**	**Total**	**Mean**	**SD**	**Total**	**means and 95% CI**
**With RT**							
Mori 2018 [24]	1.1	6.15	25	0.4	7.2	25	0.1045 [-0.450, 0.659]
Ashley 2021 [25]	-1.281	7.699	28	-0.37	8.000	39	-0.116 [-0.602, 0.370]
**Without RT**							
Zhu 2015 [34]	0.6	2.11	101	1.05	2.24	95	-0.207 [-0.488, 0.074]
Hodgson 2012 [36]	0	11.4	96	0.9	11.6	92	-0.078 [-0.364, 0.208]

RT = resistance training; SD = standard deviation; Std diff = standard difference; CI = confidence interval

Supplemental Table S5. Differences in daily nutrients intake between whey protein supplements and control supplements considering the subgroup analysis of RT/non-RT

Protein intake (g/day)

Study	Whey protein Mean	Whey protein SD	Whey protein Total	Control Mean	Control SD	Control Total	Std diff in means and 95% CI
**With RT**							
Sugihara 2018 [30]	24.86	8.34	15	4.143	9.9	16	2.257 [1.356, 3.158]
**Without RT**							
Hodgson 2012 [36]	-12	18.5	96	-2	16.5	92	-0.204 [-0.635, 0.227]
Stojkovic 2017 [33]	-5.1	17.88	38	-1.7	15.6	46	-0.5695 [-0.861, -0.278]

Carbohydrate intake (g/day)

Study	Whey protein Mean	Whey protein SD	Whey protein Total	Control Mean	Control SD	Control Total	Std diff in means and 95% CI
**With RT**							
Sugihara 2018 [30]	-13.1	26.92	15	-6.74	33.99	16	-0.2065 [-0.913, 0.500]
**Without RT**							
Hodgson 2012 [36]	-10	49.1	96	-20	46.52	92	0.209 [-0.078, 0.496]
Stojkovic 2017 [33]	-8.6	51.78	38	1.6	50.87	46	-0.199 [-0.630, 0.232]

Supplemental Table S5. Cont.

Fat intake (g/day)

Study	Whey protein Mean	Whey protein SD	Whey protein Total	Control Mean	Control SD	Control Total	Std diff in means and 95% CI
**With RT**							
Sugihara 2018 [30]	2.7143	8.8	15	1.3	8.25	16	0.166 [-0.540, 0.872]
**Without RT**							
Hodgson 2012 [36]	-1	21.93	96	3	21.65	92	-0.1835 [-0.470, 0.103]
Stojkovic 2017 [33]	-4.4	19.73	38	-5.4	25.09	46	0.044 [-0.386, 0.474]

Total energy intake (g/day)

Study	Whey protein Mean	Whey protein SD	Whey protein Total	Control Mean	Control SD	Control Total	Std diff in means and 95% CI
**With RT**							
Sugihara 2018 [30]	71.13	145.8	15	1.54	178.4	16	0.4255 [-0.287, 1.138]
**Without RT**							
Hodgson 2012 [36]	-120.68	416	96	-68.17	380	92	-0.1015 [-0.531, 0.328]
Stojkovic 2017 [33]	-70.4	329.8	38	-34.5	372.35	46	-0.1315 [-0.418, 0.155]

RT = resistance training; SD = standard deviation; Std diff = standard difference; CI = confidence interval

Supplemental Figure S1. Forest plot of the grip strength

Group 1.00: with resistance training; Group 2.00: without resistance training

Group by group	Study name	Std diff in means	Standard error	Variance	Lower limit	Upper limit	Z-Value	p-Value
		Statistics for each study						
1.00	Mori et al. 2018	0.375	0.285	0.081	-0.185	0.934	1.313	0.189
1.00	Kirk et al. 2020 (w/ ex)	0.169	0.401	0.161	-0.617	0.955	0.421	0.674
1.00	Ashley et al. 2021	0.030	0.248	0.061	-0.456	0.515	0.120	0.905
1.00		0.176	0.170	0.029	-0.156	0.509	1.040	0.298
2.00	Zhu et al. 2015 (1yr data)	0.061	0.144	0.021	-0.221	0.343	0.424	0.672
2.00	Kirk et al. 2020 (w/o ex)	-0.023	0.408	0.167	-0.823	0.777	-0.057	0.955
2.00		0.052	0.136	0.018	-0.215	0.318	0.381	0.703
Overall		0.100	0.106	0.011	-0.107	0.308	0.948	0.343

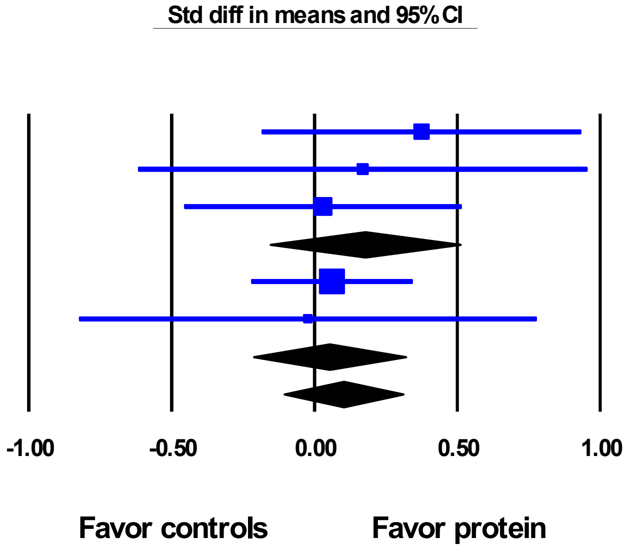

Supplemental Figure S2. Forest plot of the biceps curl strength

Study name	Std diff in means	Standard error	Variance	Lower limit	Upper limit	Z-Value	p-Value
Weisgarber et al. 2014	0.522	0.455	0.207	-0.369	1.413	1.148	0.251
Nabuco et al. 2018 (B, 12 week)	0.755	0.312	0.098	0.143	1.367	2.418	0.016
	0.680	0.257	0.066	0.176	1.185	2.642	0.008

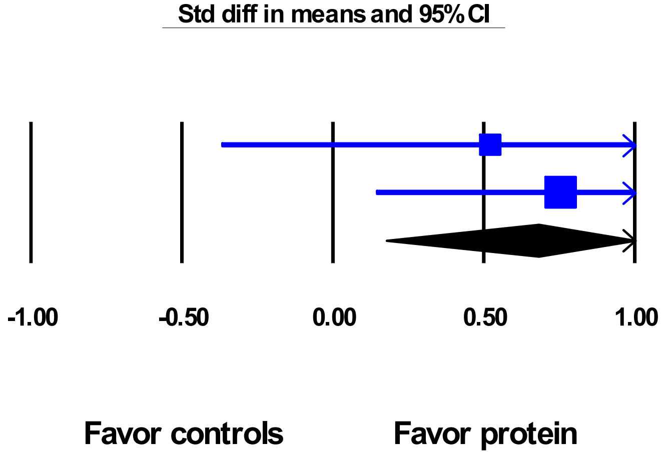

Supplemental Figure S3. Forest plot of the knee extension strength

Group 1.00: with resistance training; Group 2.00: without resistance training

Group by group	Study name	Std diff in means	Standard error	Variance	Lower limit	Upper limit	Z-Value	p-Value
1.00	Weisgarber et al. 2014	0.021	0.447	0.200	-0.855	0.898	0.047	0.962
1.00	Mori et al. 2018	0.647	0.290	0.084	0.078	1.215	2.229	0.026
1.00	Nabuco et al. 2018 (B, 12 week)	0.000	0.302	0.091	-0.592	0.592	0.000	1.000
1.00	Kirk et al. 2020 (w/ ex)	0.773	0.415	0.172	-0.040	1.586	1.862	0.063
1.00		0.364	0.202	0.041	-0.031	0.759	1.804	0.071
2.00	Zhu et al. 2015 (1yr data)	-0.126	0.144	0.021	-0.409	0.156	-0.875	0.382
2.00	Kirk et al. 2020 (w/o ex)	-0.124	0.409	0.167	-0.925	0.676	-0.305	0.761
2.00		-0.126	0.136	0.018	-0.392	0.141	-0.926	0.354
Overall		0.027	0.113	0.013	-0.194	0.248	0.240	0.810

Std diff in means and 95%CI

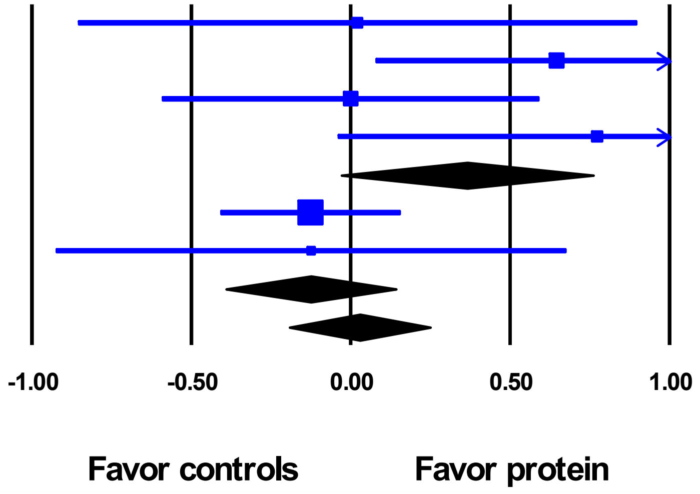

Supplemental Figure S4. Forest plot of the knee flexion strength

Group 1.00: with resistance training; Group 2.00: without resistance training

Group by group	Study name	Std diff in means	Standard error	Variance	Lower limit	Upper limit	Z-Value	p-Value
		Statistics for each study						
1.00	Weisgarber et al. 2014	-0.257	0.449	0.202	-1.137	0.623	-0.572	0.567
1.00	Kirk et al. 2020 (w/ ex)	0.812	0.416	0.173	-0.005	1.628	1.949	0.051
1.00		0.291	0.534	0.285	-0.756	1.337	0.544	0.586
2.00	Zhu et al. 2015 (1yr data)	0.041	0.144	0.021	-0.241	0.324	0.286	0.775
2.00	Kirk et al. 2020 (w/o ex)	-0.074	0.408	0.167	-0.874	0.726	-0.181	0.856
2.00		0.028	0.136	0.018	-0.238	0.295	0.210	0.834
Overall		0.044	0.132	0.017	-0.214	0.302	0.337	0.736

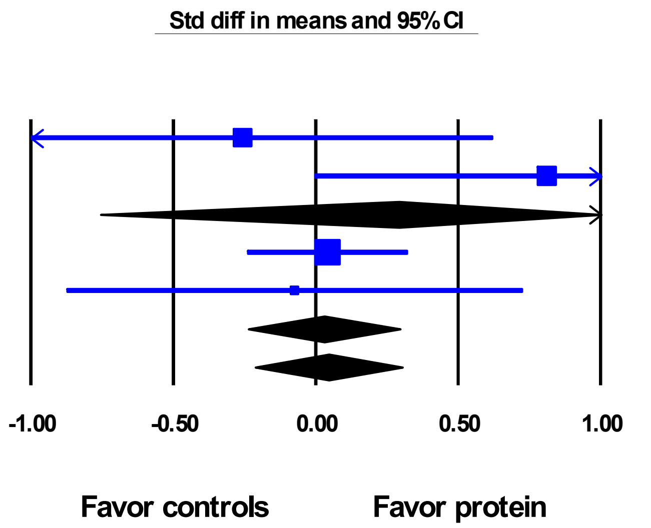

Supplemental Figure S5. Forest plot of the gait speed

Study name	Std diff in means	Standard error	Variance	Lower limit	Upper limit	Z-Value	p-Value
Mori et al. 2018	0.000	0.283	0.080	-0.554	0.554	0.000	1.000
Nabuco et al. 2018 (B, 12 week)	0.642	0.309	0.096	0.036	1.249	2.076	0.038
Ashley et al. 2021	-0.072	0.248	0.061	-0.557	0.414	-0.289	0.773
	0.163	0.216	0.047	-0.261	0.586	0.752	0.452

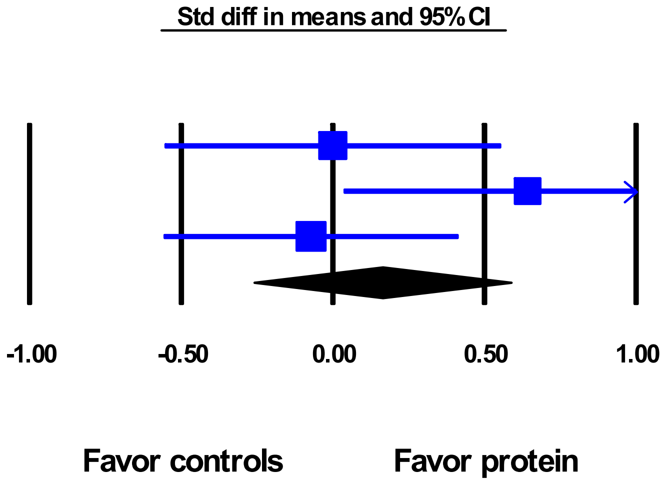

Supplemental Figure S6. Forest plot of the chest press

Study name	Std diff in means	Standard error	Variance	Lower limit	Upper limit	Z-Value	p-Value
Nabuco et al. 2018 (B, 12 week)	0.114	0.302	0.091	-0.478	0.706	0.378	0.706
Ashley et al. 2021	0.104	0.248	0.061	-0.382	0.590	0.419	0.675
	0.108	0.192	0.037	-0.268	0.484	0.564	0.573

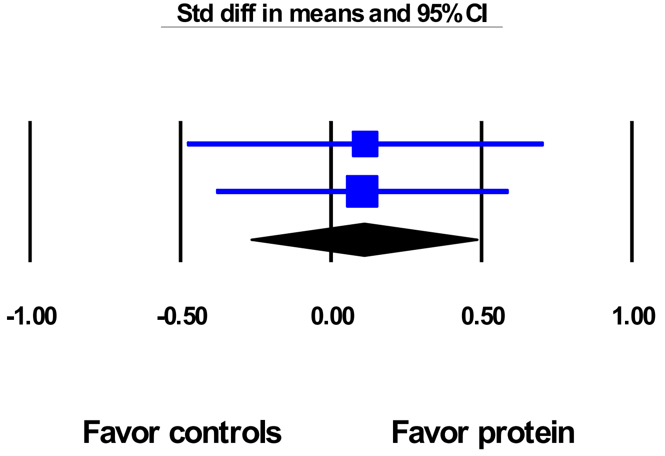

Supplemental Figure S7. Forest plot of the rising from sitting position

Group 1.00: with resistance training; Group 2.00: without resistance training

Group by group	Study name	Std diff in means	Standard error	Variance	Lower limit	Upper limit	Z-Value	p-Value
1.00	Nabuco et al. 2018 (B, 12 week)	-0.292	0.303	0.092	-0.887	0.303	-0.962	0.336
1.00		-0.292	0.303	0.092	-0.887	0.303	-0.962	0.336
2.00	Zhu et al. 2015 (1yr data)	0.022	0.144	0.021	-0.260	0.304	0.152	0.879
2.00		0.022	0.144	0.021	-0.260	0.304	0.152	0.879
Overall		-0.036	0.130	0.017	-0.291	0.219	-0.275	0.783

Statistics for each study

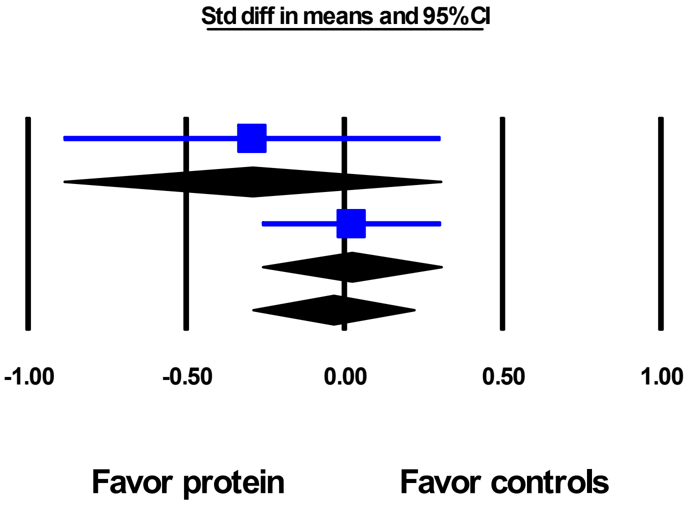

Supplemental Figure S8. Forest plot of the upper limb lean mass

Group 1.00: with resistance training; Group 2.00: without resistance training

Group by group	Study name	Std diff in means	Standard error	Variance	Lower limit	Upper limit	Z-Value	p-Value
1.00	Mori et al. 2018	0.000	0.283	0.080	-0.554	0.554	0.000	1.000
1.00	Nabuco et al. 2018 (B, 12 week)	0.000	0.302	0.091	-0.592	0.592	0.000	1.000
1.00		0.000	0.206	0.043	-0.405	0.405	0.000	1.000
2.00	Zhu et al. 2015 (1yr data)	-0.359	0.144	0.021	-0.641	-0.076	-2.489	0.013
2.00		-0.359	0.144	0.021	-0.641	-0.076	-2.489	0.013
Overall		-0.241	0.118	0.014	-0.473	-0.010	-2.041	0.041

Statistics for each study

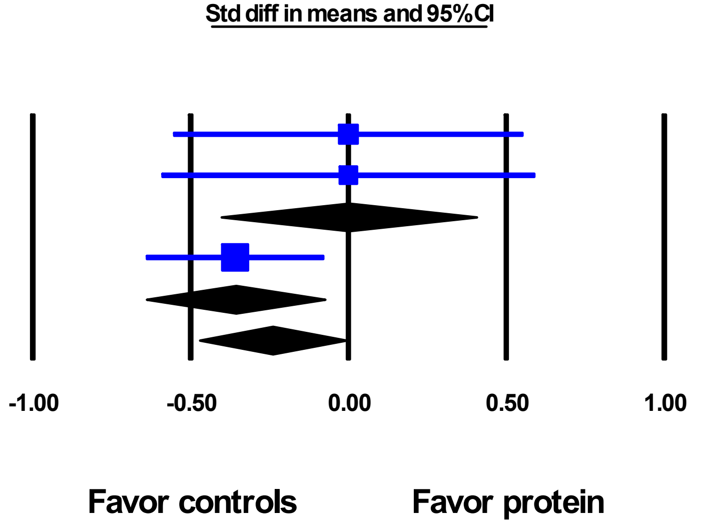

Supplemental Figure S9. Forest plot of the lower limb lean mass

Group 1.00: with resistance training; Group 2.00: without resistance training

Group by group	Study name	Std diff in means	Standard error	Variance	Lower limit	Upper limit	Z-Value	p-Value
		Statistics for each study						
1.00	Mori et al. 2018	0.887	0.296	0.088	0.306	1.468	2.992	0.003
1.00	Nabuco et al. 2018 (B, 12 week)	1.370	0.335	0.112	0.713	2.028	4.087	0.000
1.00		1.103	0.240	0.058	0.632	1.574	4.589	0.000
2.00	Zhu et al. 2015 (1yr data)	-0.359	0.144	0.021	-0.641	-0.076	-2.489	0.013
2.00		-0.359	0.144	0.021	-0.641	-0.076	-2.489	0.013
Overall		0.028	0.124	0.015	-0.214	0.270	0.224	0.823

Std diff in means and 95%CI

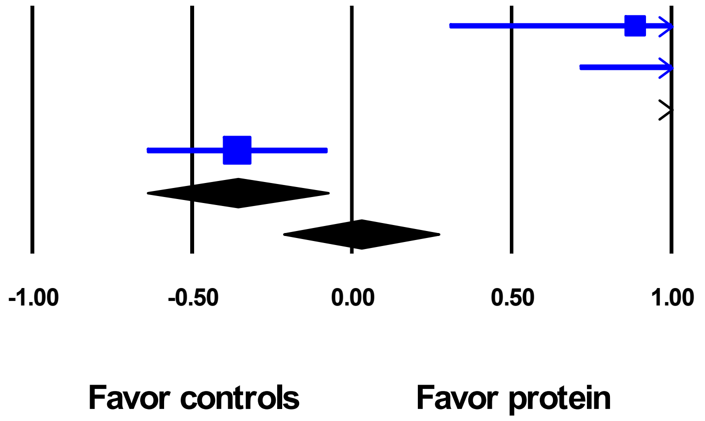

Supplemental Figure S10. Forest plot of the skeletal muscle mass

Group 1.00: with resistance training; Group 2.00: without resistance training

Group by group	Study name	Std diff in means	Standard error	Variance	Lower limit	Upper limit	Z-Value	p-Value
1.00	Nabuco et al. 2018 (B, 12 week)	0.933	0.318	0.101	0.310	1.556	2.936	0.003
1.00	Kirk et al. 2020 (w/ ex)	-0.039	0.400	0.160	-0.824	0.746	-0.098	0.922
1.00		0.478	0.485	0.235	-0.473	1.428	0.984	0.325
2.00	Kirk et al. 2020 (w/o ex)	-0.202	0.409	0.167	-1.004	0.600	-0.494	0.621
2.00		-0.202	0.409	0.167	-1.004	0.600	-0.494	0.621
Overall		0.080	0.313	0.098	-0.533	0.693	0.257	0.797

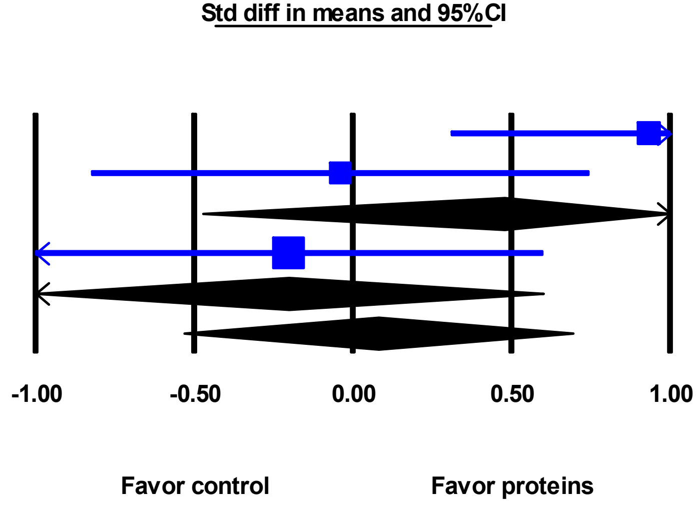

Supplemental Figure S11. Forest plot of the fat mass

Group 1.00: with resistance training; Group 2.00: without resistance training

Group by group	Study name	Std diff in means	Standard error	Variance	Lower limit	Upper limit	Z-Value	p-Value
1.00	Kirk et al. 2020 (w/ ex)	0.022	0.400	0.160	-0.763	0.806	0.054	0.957
1.00	Nabuco et al. 2019 (2)	-0.063	0.302	0.091	-0.655	0.529	-0.209	0.835
1.00	Ashley et al. 2021	-0.148	0.248	0.062	-0.634	0.338	-0.597	0.551
1.00		-0.088	0.173	0.030	-0.427	0.250	-0.512	0.609
2.00	Kirk et al. 2020 (w/o ex)	0.105	0.409	0.167	-0.695	0.906	0.258	0.796
2.00	Stojkovic et al. 2017	-2.642	0.299	0.090	-3.229	-2.055	-8.826	0.000
2.00		-1.282	1.374	1.887	-3.975	1.410	-0.934	0.351
Overall		-0.107	0.172	0.029	-0.443	0.229	-0.624	0.532

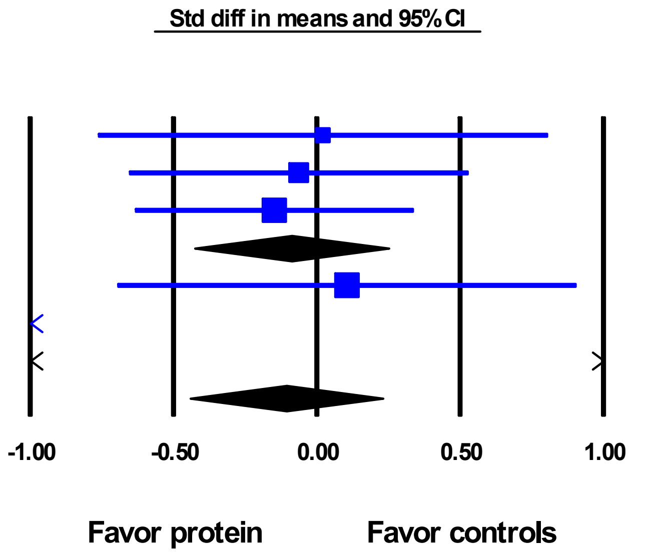

Supplemental Figure S12. Forest plot of the body weight

Group 1.00: with resistance training; Group 2.00: without resistance training

Group by group	Study name	Std diff in means	Standard error	Variance	Lower limit	Upper limit	Z-Value	p-Value
1.00	Mori et al. 2018	0.105	0.283	0.080	-0.450	0.659	0.369	0.712
1.00	Ashley et al. 2021	-0.116	0.248	0.061	-0.602	0.370	-0.467	0.641
1.00		-0.020	0.186	0.035	-0.386	0.345	-0.108	0.914
2.00	Zhu et al. 2015 (1yr data)	-0.207	0.143	0.021	-0.488	0.074	-1.444	0.149
2.00	Hodgson et al. 2012 (1yr data)	-0.078	0.146	0.021	-0.364	0.208	-0.536	0.592
2.00		-0.144	0.102	0.010	-0.344	0.057	-1.406	0.160
Overall		-0.115	0.090	0.008	-0.291	0.061	-1.285	0.199

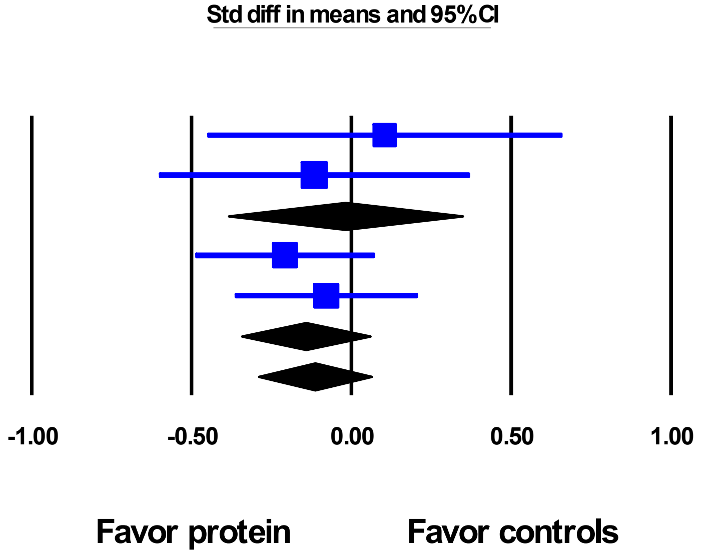

Supplemental Figure S13. Forest plot of the total energy intake

Group 1.00: with resistance training; Group 2.00: without resistance training

Group by group	Study name	Std diff in means	Standard error	Variance	Lower limit	Upper limit	Z-Value	p-Value
1.00	Sugihara et al. 2018	0.426	0.363	0.132	-0.287	1.138	1.171	0.241
1.00		0.426	0.363	0.132	-0.287	1.138	1.171	0.241
2.00	Stojkovic et al. 2017	-0.101	0.219	0.048	-0.531	0.328	-0.463	0.644
2.00	Hodgson et al. 2012 (1yr data)	-0.132	0.146	0.021	-0.418	0.155	-0.902	0.367
2.00		-0.122	0.122	0.015	-0.361	0.116	-1.007	0.314
Overall		-0.067	0.115	0.013	-0.293	0.159	-0.583	0.560

Std diff in means and 95%CI

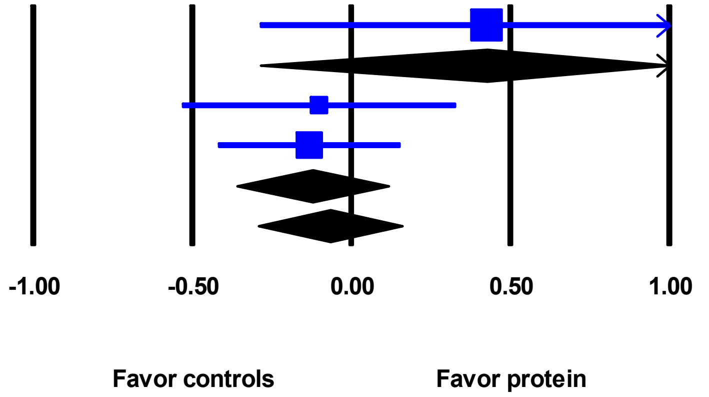

Supplemental Figure S14. Forest plot of the carbohydrate intake

Group 1.00: with resistance training; Group 2.00: without resistance training

Group by group	Study name	Std diff in means	Standard error	Variance	Lower limit	Upper limit	Z-Value	p-Value
1.00	Sugihara et al. 2018	-0.207	0.360	0.130	-0.913	0.500	-0.573	0.566
1.00		-0.207	0.360	0.130	-0.913	0.500	-0.573	0.566
2.00	Hodgson et al. 2012 (1yr data)	0.209	0.146	0.021	-0.078	0.496	1.428	0.153
2.00	Stojkovic et al. 2017	-0.199	0.220	0.048	-0.630	0.232	-0.905	0.365
2.00		0.038	0.201	0.041	-0.356	0.432	0.189	0.850
Overall		-0.020	0.176	0.031	-0.365	0.324	-0.115	0.909

Std diff in means and 95%CI

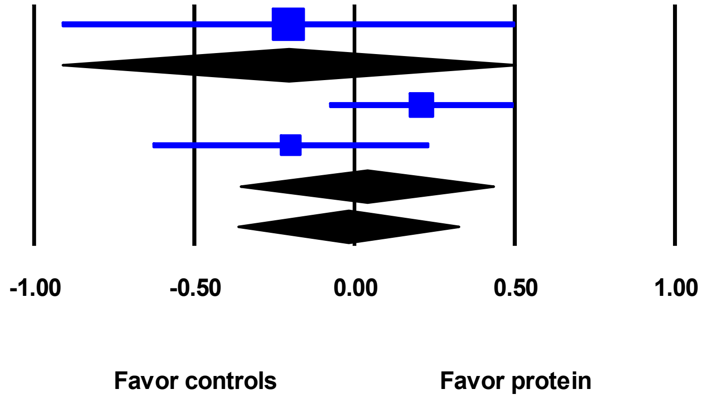

Supplemental Figure S15. Forest plot of the fat intake

Group 1.00: with resistance training; Group 2.00: without resistance training

Group by group	Study name	Std diff in means	Standard error	Variance	Lower limit	Upper limit	Z-Value	p-Value
1.00	Sugihara et al. 2018	0.166	0.360	0.130	-0.540	0.872	0.461	0.645
1.00		0.166	0.360	0.130	-0.540	0.872	0.461	0.645
2.00	Hodgson et al. 2012 (1yr data)	-0.184	0.146	0.021	-0.470	0.103	-1.255	0.209
2.00	Stojkovic et al. 2017	0.044	0.219	0.048	-0.386	0.474	0.200	0.842
2.00		-0.114	0.122	0.015	-0.352	0.125	-0.934	0.351
Overall		-0.085	0.115	0.013	-0.311	0.141	-0.737	0.461

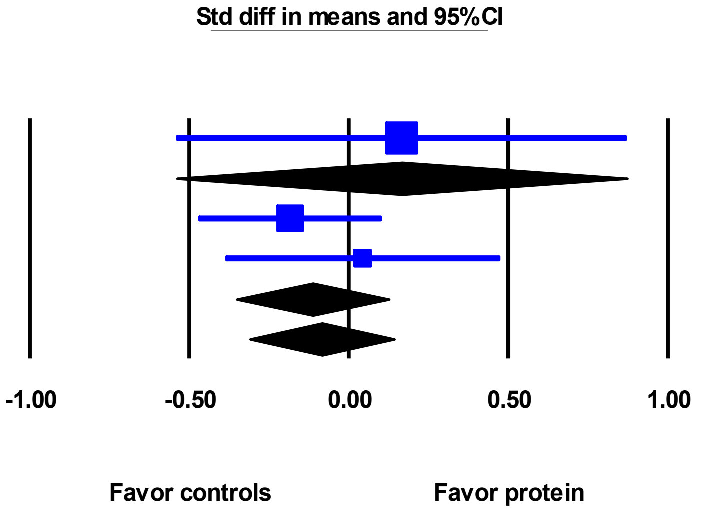

Supplemental Figure S16. Forest plot of the protein intake

Group 1.00: with resistance training; Group 2.00: without resistance training

Group by group	Study name	Std diff in means	Standard error	Variance	Lower limit	Upper limit	Z-Value	p-Value
		Statistics for each study						
1.00	Sugihara et al. 2018	2.257	0.460	0.211	1.356	3.158	4.909	0.000
1.00		2.257	0.460	0.211	1.356	3.158	4.909	0.000
2.00	Stojkovic et al. 2017	-0.204	0.220	0.048	-0.635	0.227	-0.928	0.353
2.00	Hodgson et al. 2012 (1yr data)	-0.570	0.149	0.022	-0.861	-0.278	-3.829	0.000
2.00		-0.423	0.179	0.032	-0.774	-0.071	-2.356	0.018
Overall		-0.069	0.167	0.028	-0.396	0.259	-0.410	0.682

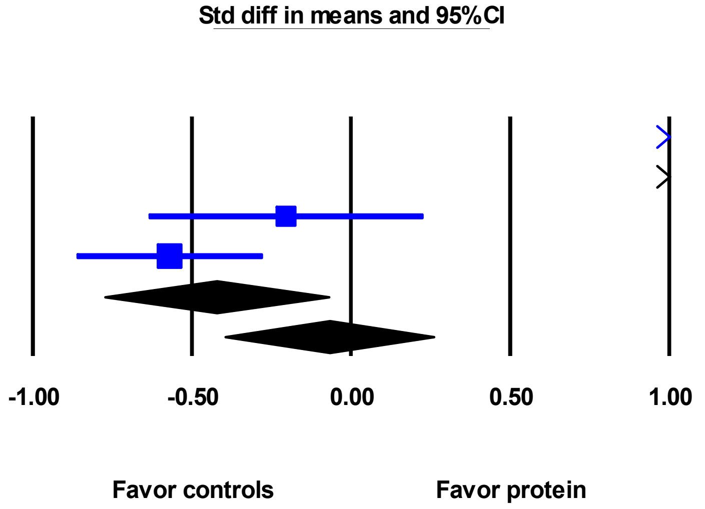

Supplement: Supplementary file 1 [file nutrients-14-04210-s001.zip › nutrients-1893879-supplementary.pdf]
